# Supplementary material for: Attenuation of IFITM proteins’ antiviral activity through sequestration into intraluminal vesicles of late endosomes
Source: Front Immunol. 2025 Aug 21;16:1647166. doi: 10.3389/fimmu.2025.1647166 (PMC12409171; doi:10.3389/fimmu.2025.1647166)
Supplement: Supplementary file 1 [file DataSheet1.pdf]

Supplementary Material

1 Supplementary Figures and Tables

1.1 Supplementary Figures

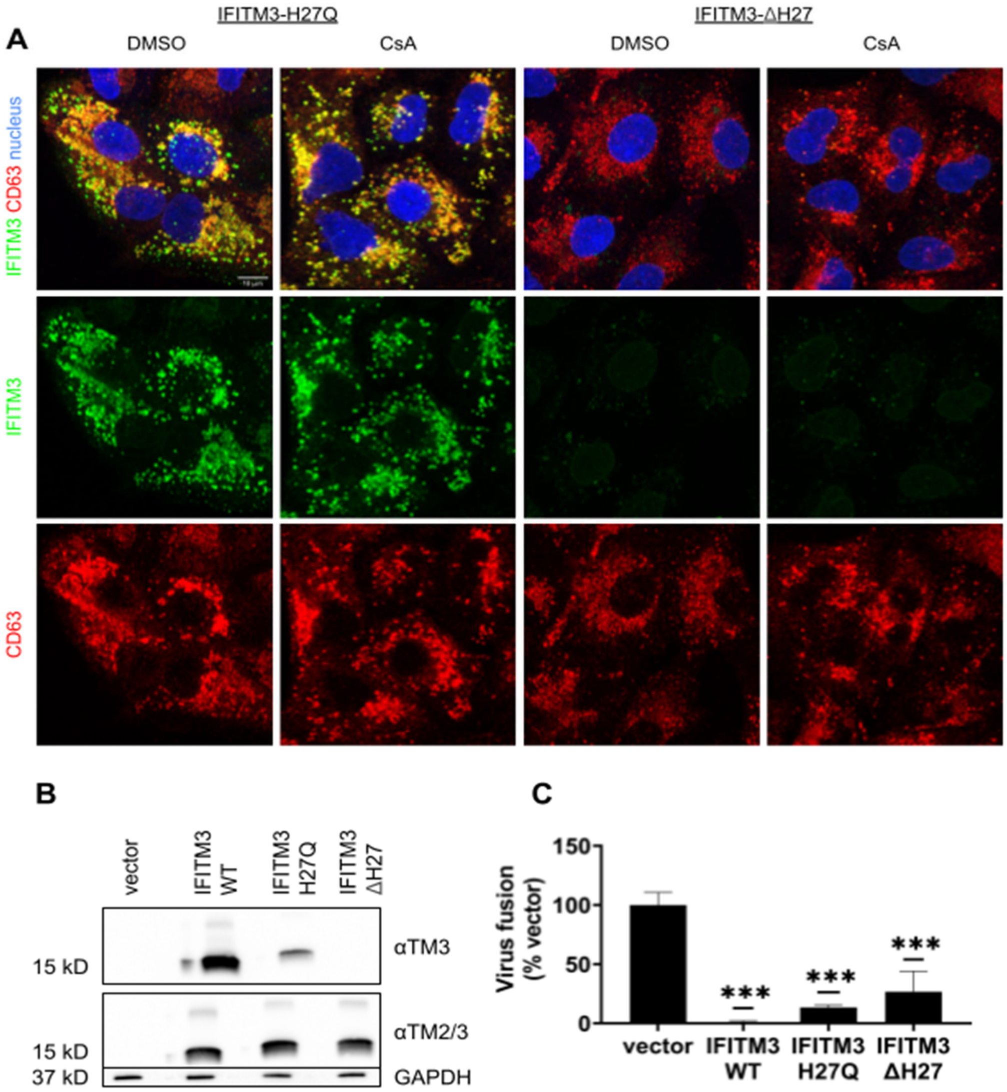

Figure S1. Epitope mapping for EPR5242 anti-IFITM3 antibody. (A) A549 cells were stably transduced with the IFITM3 mutants, H27Q or ΔH27, fixed, permeabilized with TX-100, stained using the anti-IFITM3 EPR5242 antibody and with anti-CD63 antibody, and imaged on a confocal microscope. (B) A549 cells transduced with empty vector, wild-type IFITM3 or the H27Q or ΔH27 IFITM3 mutants we harvested, lysed and analyzed by Western blotting. Two different anti-IFITM3 antibodies were used: the recombinant rabbit EPR5242 (presumably, against the N-terminus of IFITM3) and the monoclonal mouse 2E8D12 (against an unknown IFITM2/3 epitope). (C)

Transduced cells from (B) were challenged with IAVpp, and virus-cell fusion was measured using a BlaM assay. Data are means and SD from a triplicate measurement.

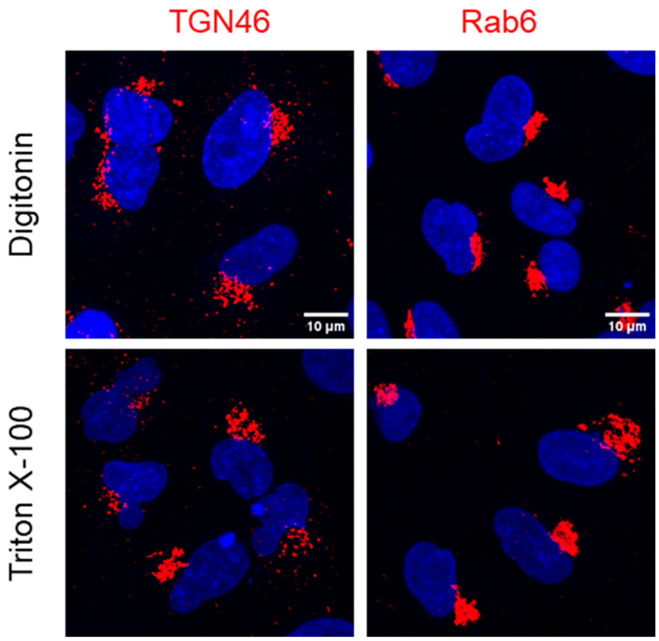

Figure S2. TGN46 and Rab6 subcellular localization in cells permeabilized by digitonin or Triton X-100. A549.IFITM3 cells were fixed, permeabilized with either digitonin or TX-100, and stained with either anti-TGN46 or anti-Rab6 antibodies.

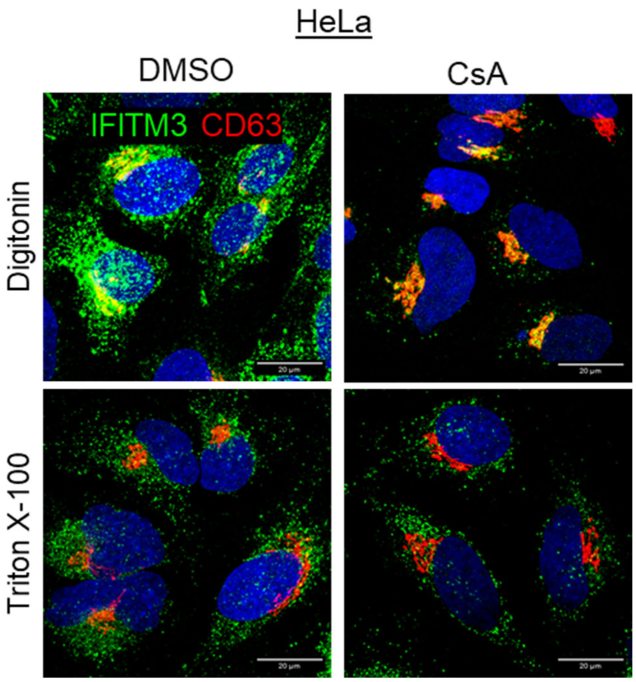

Figure S3. Different permeabilization protocols of HeLa cells revealed two pools of IFITM3. HeLa cells were incubated in the presence or absence of CsA (20 μM) for 90 minutes, fixed, permeabilized with either digitonin or TX-100 and stained with anti-IFITM3 and anti-GM130 antibodies.

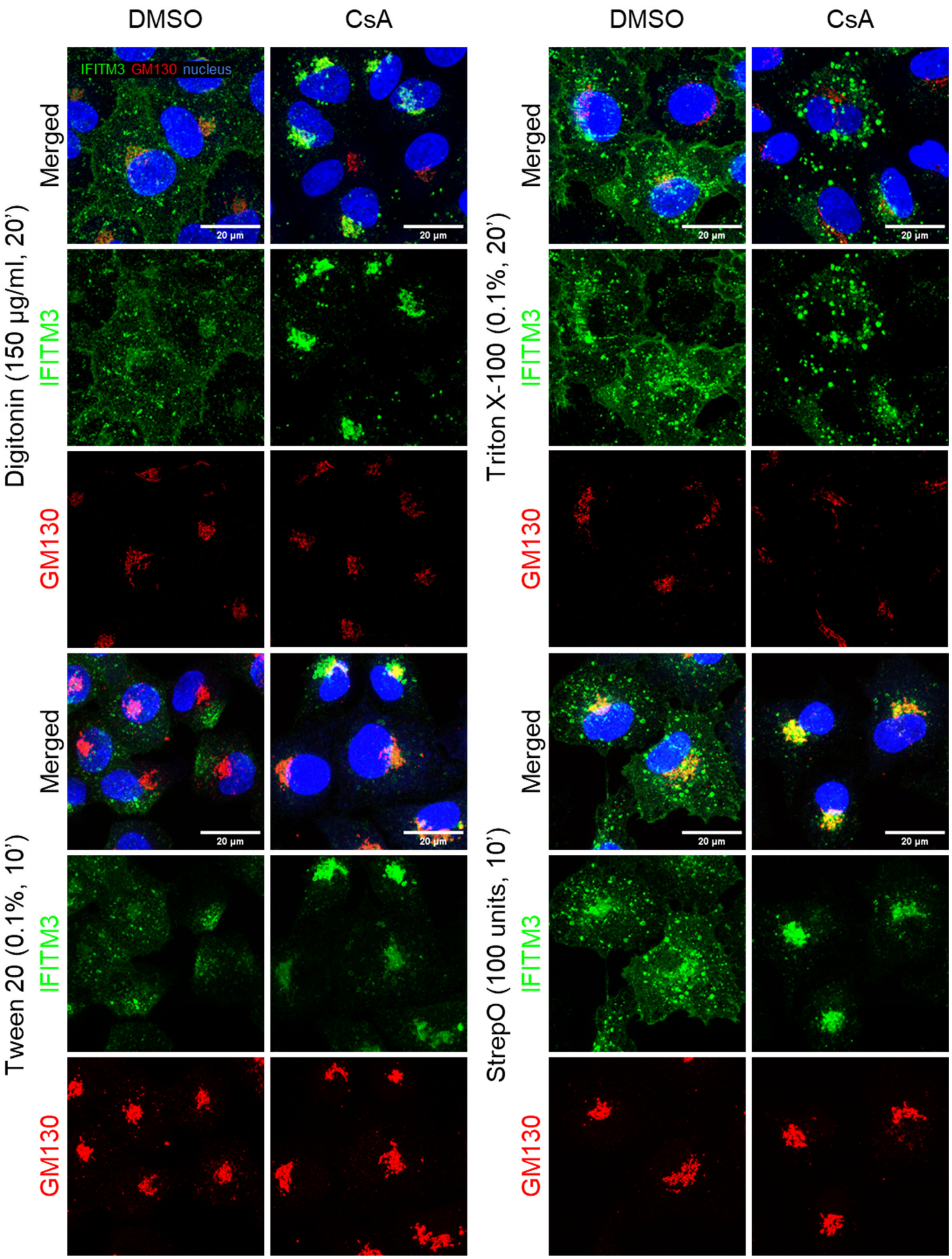

Figures S4. Subcellular distribution of IFITM3 in control and CsA treated cells following different A549.IFITM3 cell permeabilization protocols. A549.IFITM3 cells were incubated in the presence or absence of CsA (20 μM) for 90 minutes, fixed with PFA, and permeabilized with different reagents, as indicated, followed with staining using anti-IFITM3 and anti-GM130 antibodies.

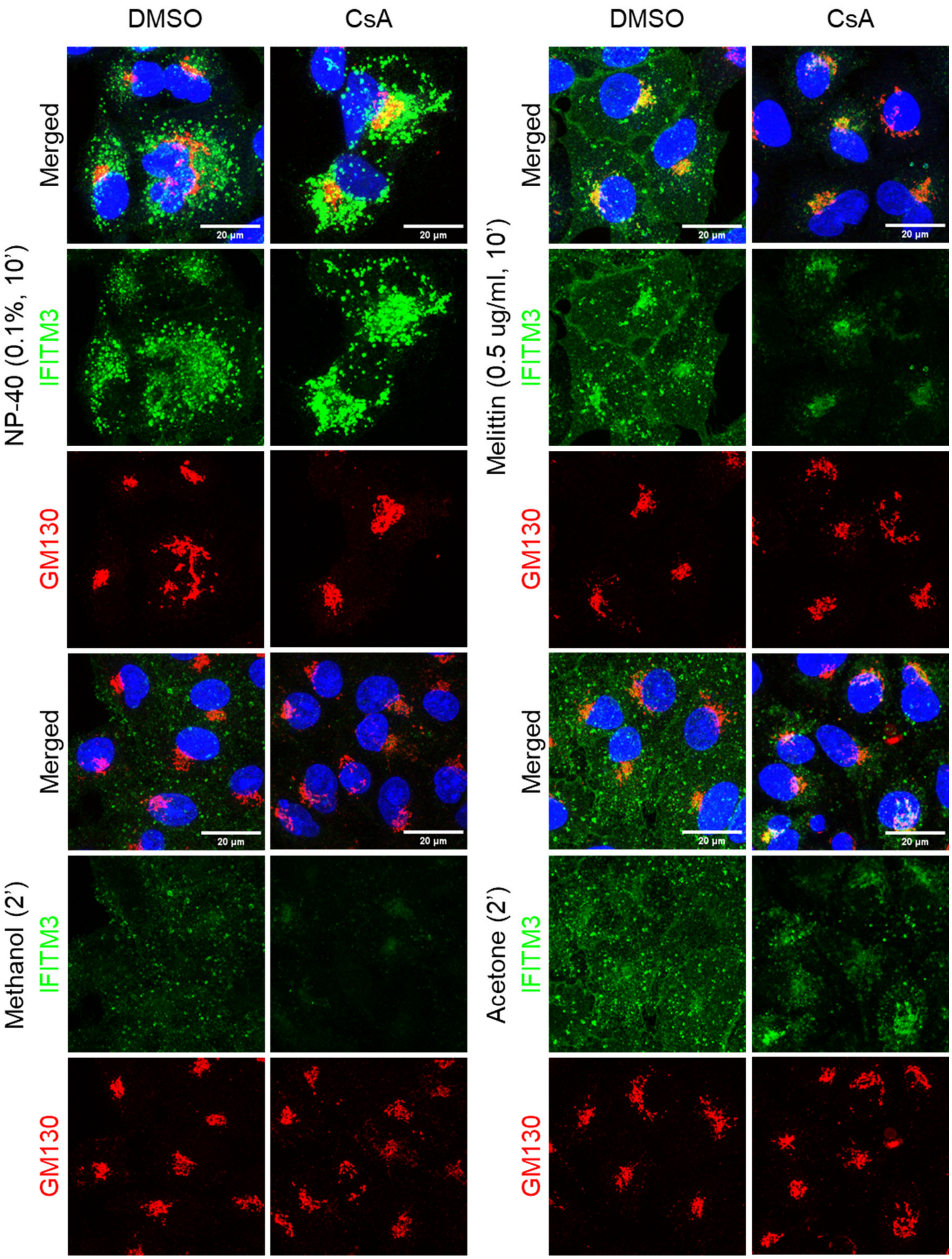

Figures S5. Subcellular distribution of IFITM3 in control and CsA treated cells following different A549.IFITM3 cell permeabilization protocols. A549.IFITM3 cells were incubated in the presence or absence of CsA (20 µM) for 90 minutes, fixed with PFA, and permeabilized with different reagents, as indicated, followed with staining using anti-IFITM3 and anti-GM130 antibodies.

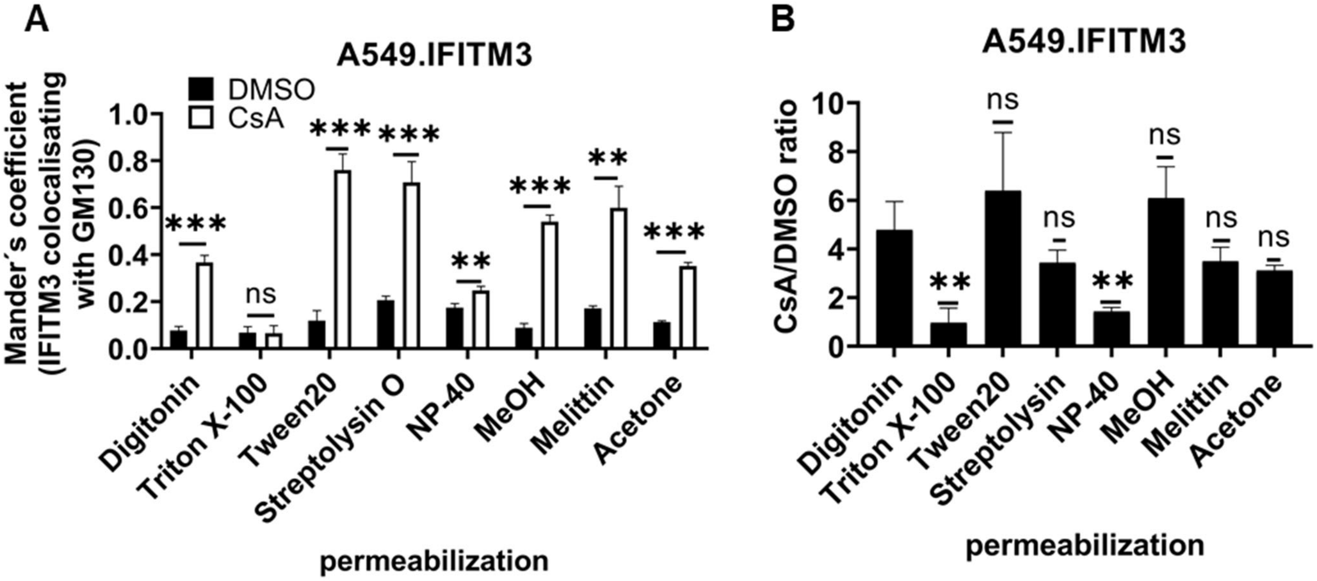

Figures S6. Analysis of IFITM3 and GM130 colocalization in A549.IFITM3 cells permeabilized with different reagents. (A) Colocalization (Mander's coefficient) of IFITM3 and GM130 for cells treated with DMSO or CsA, as well as the ratio between colocalization in DMSO and CsA-treated (see Figs. S3 and S4) was calculated using the JaCoP FiJi plugin. (B) Ratios of IFITM3/GM130 colocalization in CsA vs DMSO treated cells calculated from the results in panel (A). Statistical significance of ratios between Digitonin sample and respective sample was obtained by computing the z-score. ** $p < 0.01$; *** $p < 0.001$; ns, not significant.

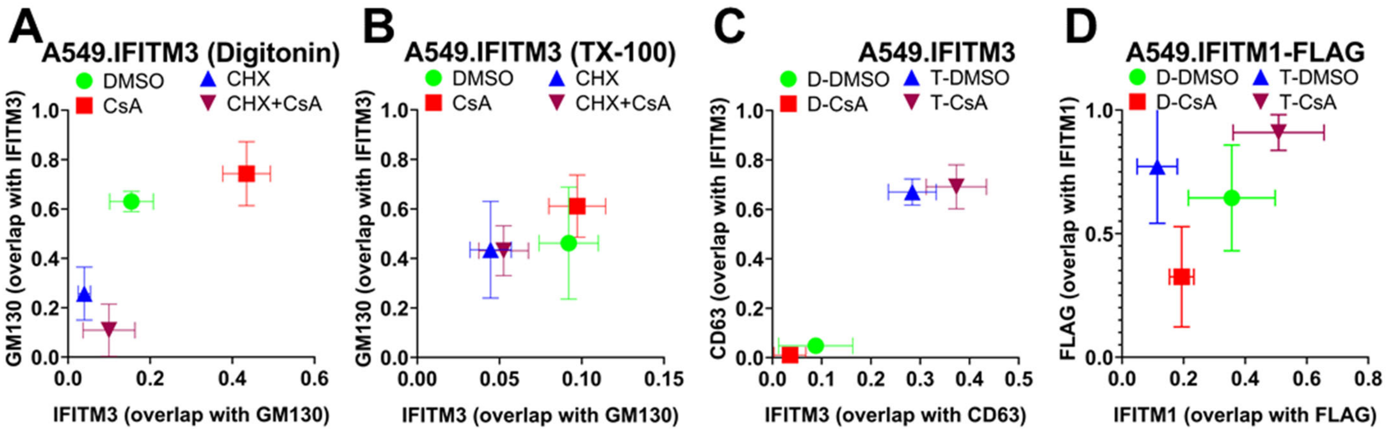

Figure S7. 3D analysis of IFITM1 and CD63 signal colocalization. Colocalization analysis of selected individual Z-stacks representing the lower half of A549.IFITM3 (A-C) or A549.IFITM1-FLAG (D) cells. Reciprocal Mander's overlap coefficients are plotted on the axis. This analysis relates to the main figures Fig. 1A, digitonin permeabilization) or Fig. 1D (TX-100 permeabilization) showing colocalization between IFITM3 and CD63 (Fig. 1G, H), and between IFITM1 and FLAG (Fig. 2C). Colocalization was determined slice-by-slice, means and error bars are the collection of data from respective slides. Data are means and S.D. of two independent experiments, each containing three fields of view. Measured Mander's Overlap Coefficients (MOC) were plotted with IFITM3 signal overlap with GM130 or CD63 on the X-axis and GM130/CD63 overlap with IFITM3 on the Y-axis.

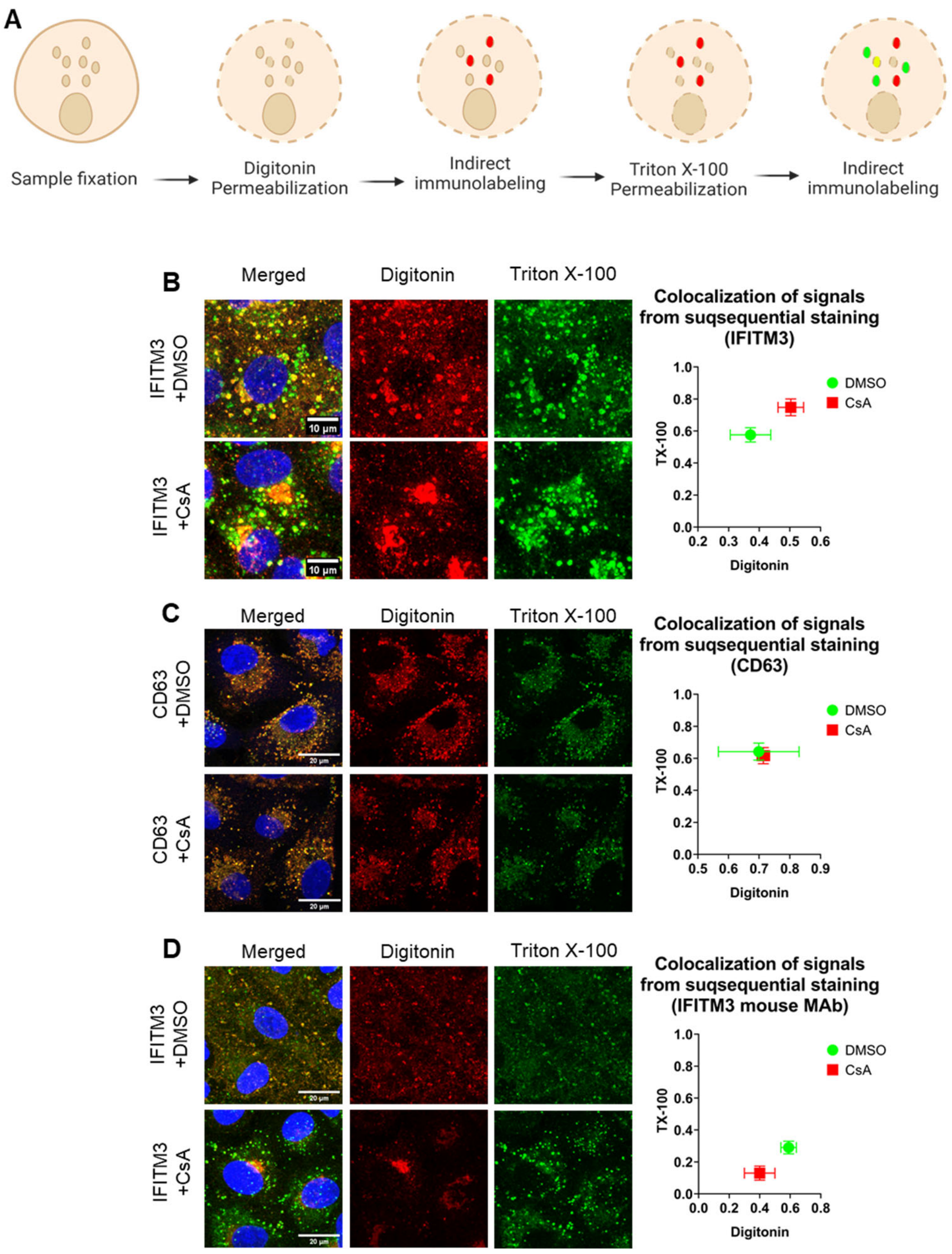

Figure S8. Distinct pools of IFITM3 revealed by different permeabilization protocols. (A) Illustration of consecutive immunostaining steps following cells permeabilization with digitonin and TX-100. (B) A549.IFITM3 cells were treated with DMSO or CsA, fixed, permeabilized with digitonin, incubated with rabbit anti-IFITM3 antibody, then permeabilized with TX-100, and incubated with mouse anti-IFITM3 antibody. Primary antibody binding was detected using different secondary antibodies conjugated to different fluorophores, to distinguish IFITM3 proteins recognized in the respective permeabilization steps. (C) A549.IFITM3 cells were treated as in (B), but an anti-CD63 antibody was used to visualize the CD63 pools accessible after each permeabilization step. (D) A549.IFITM3 cells were treated as in (B), but a mouse anti-IFITM3 antibody was used to detect IFITM3. All colocalizations of respective signals were determined by MOC on individual slices slice-by-slice (analyzed as in Fig. S6). Digitonin signals overlapping with TX-100 signals were plotted on the X axis, TX-100 signals overlapping with digitonin were plotted on the Y axis.

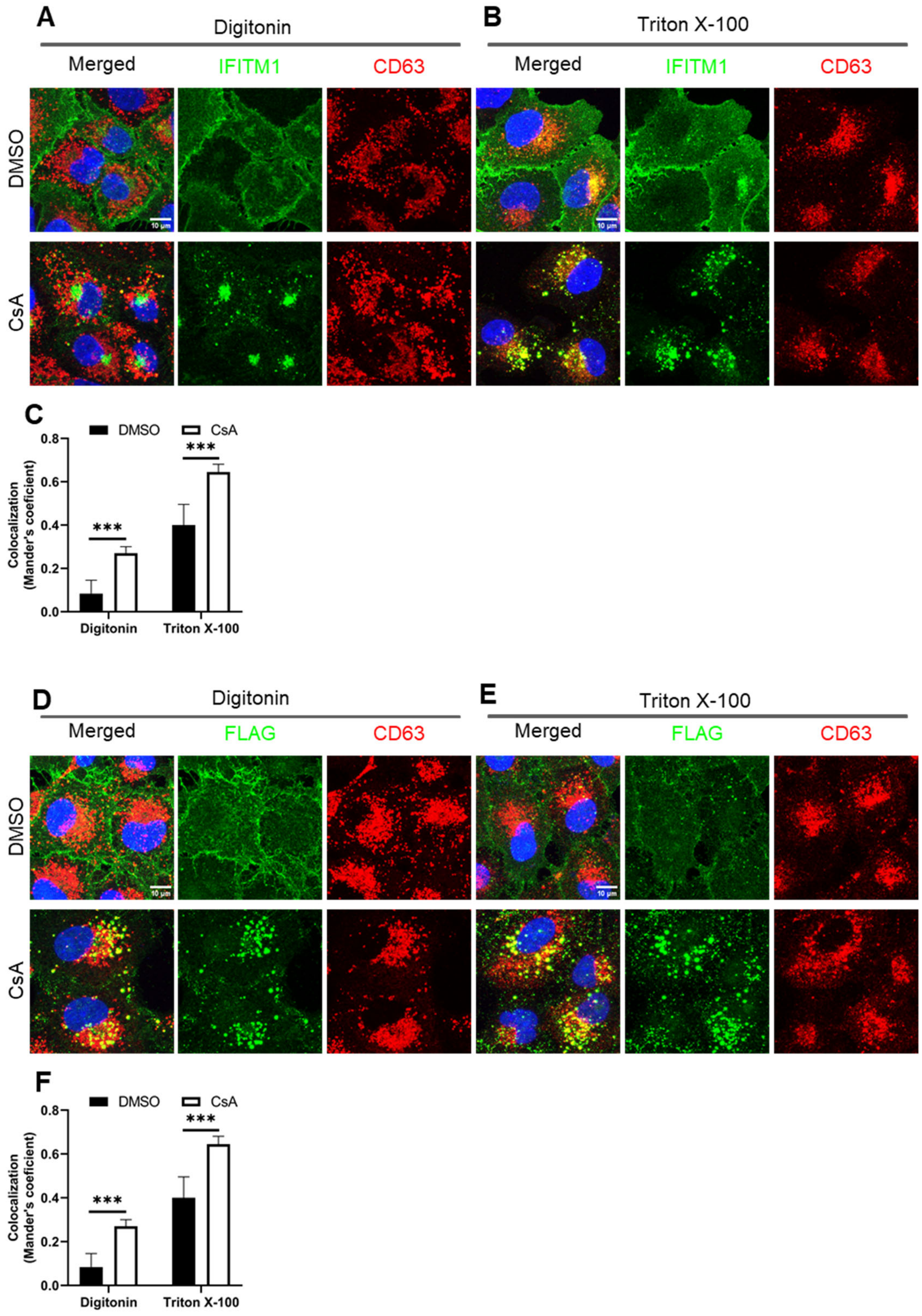

Figure S9. Disparate subcellular localizations of the IFITM1's N- and C-termini following CsA-treatment. (A, B) A549.IFITM1-FLAG cells were fixed, permeabilized with digitonin (A) or TX-100 (B), and stained for IFITM1 and CD63. (D, E) As in panels A and B, but cells were stained for FLAG

and CD63. (C, F) Colocalization of the IFITM1 N- or C-termini with CD63 under different conditions was determined for the maximum intensity projection images, using MOC.

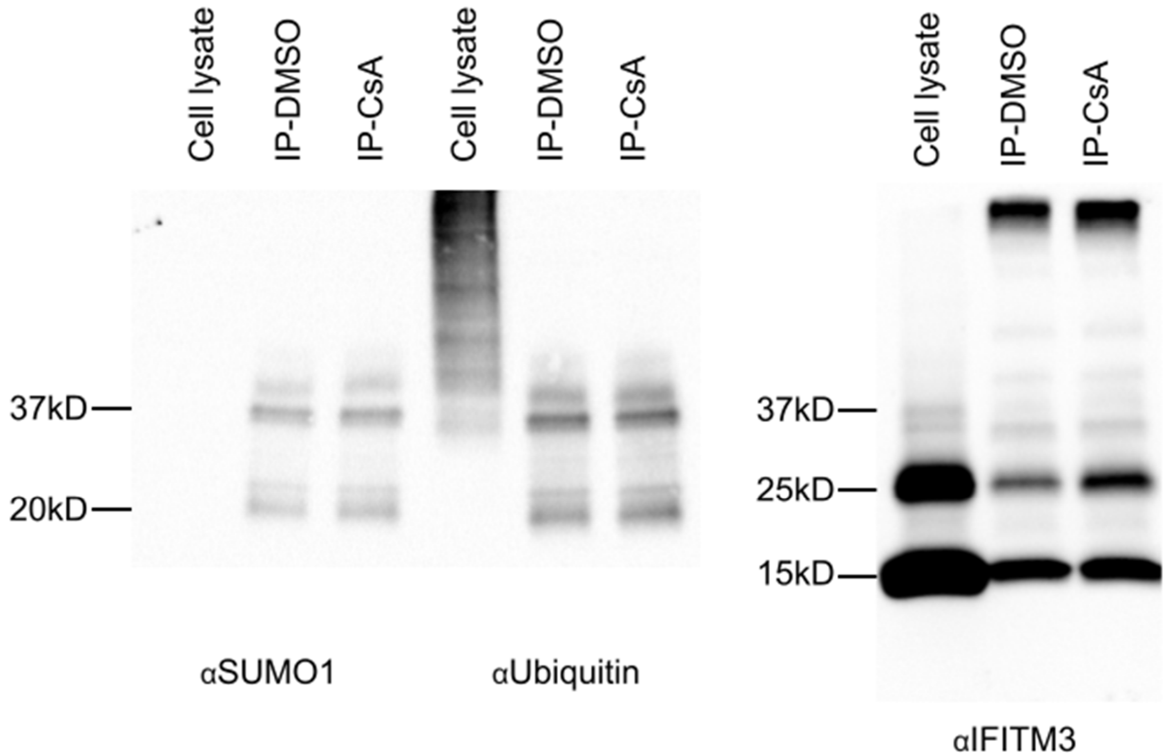

Figure S10. SUMOyalition and ubiquitination of IFITM3 in the presence of CsA is unchanged. A549.IFITM3 cells were treated for 90 min with 20 µM CsA, harvested and lysed with RIPA buffer without SDS. Lysates were subjected to pull-down using rabbit anti-IFITM3 antibodies and analyzed by Western blotting with anti-SUMO1, anti-Ubiquitin or the EPR5242 anti-IFITM3 antibodies. For details, see Materials and Methods.

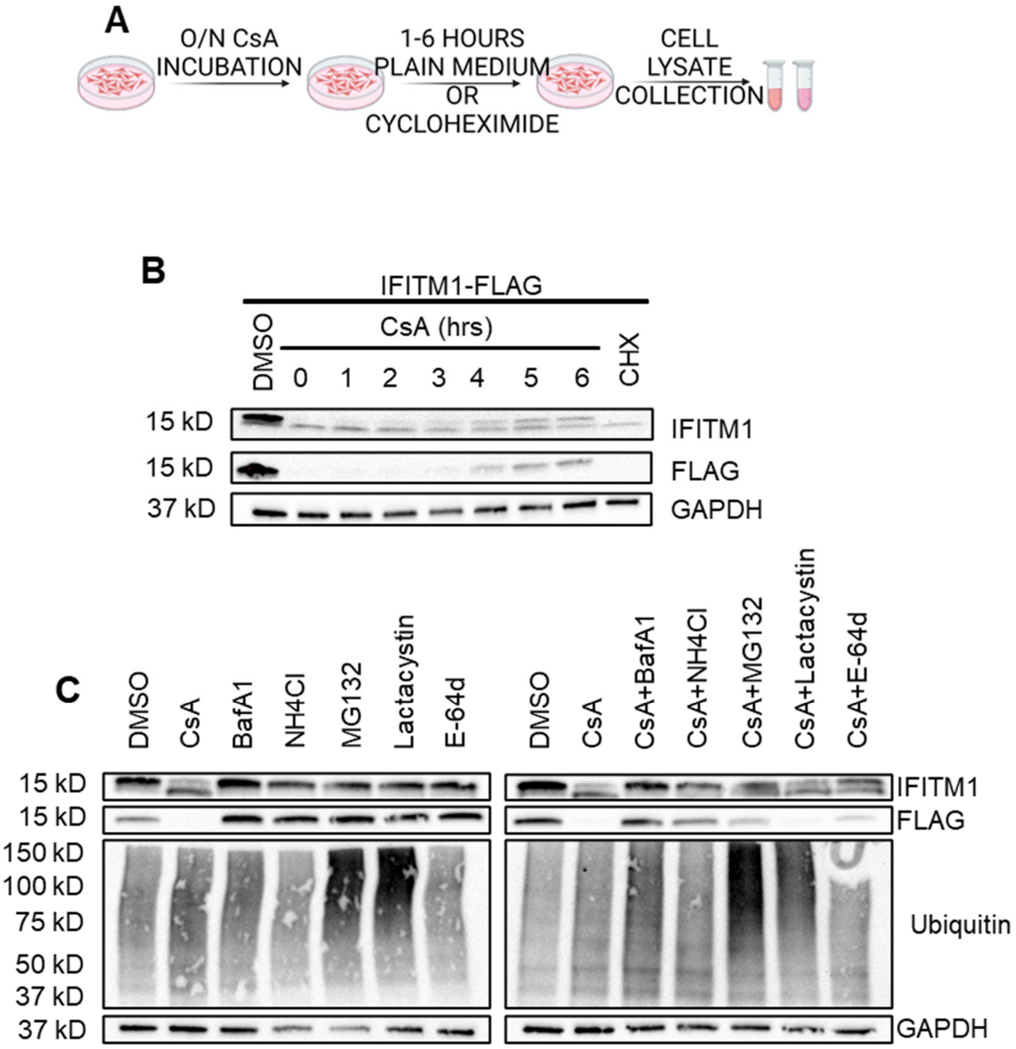

Figure S11. IFITM1 topology in CsA-treated cells is in line with the generally accepted type II topology. (A) Protocol schematics. Cells were kept with CsA in the medium overnight, washed, and incubated for the indicated times in a medium containing or lacking CHX (10 μg/mL). (B) A549.IFITM1-FLAG cells were treated as described in (A), harvested, and analyzed by Western blotting for IFITM1 and FLAG. (C) A549.IFITM1-FLAG cells were pre-incubated with inhibitors of endosome acidification, Bafilomycin A1 (BafA1, 1 μM) or ammonium chloride (NH_4Cl, 40 mM), proteasomal inhibitors, MG132 (10 μM) or Lactacystin (10 μM), or the pan-cathepsin inhibitor, E-64d (20 μM). After one hour, CsA was added to the medium, cells were incubated for 6 more hours, harvested, lysed, and examined by Western blotting using anti-IFITM1, -FLAG, -Ubiquitin, or -GAPDH antibodies.

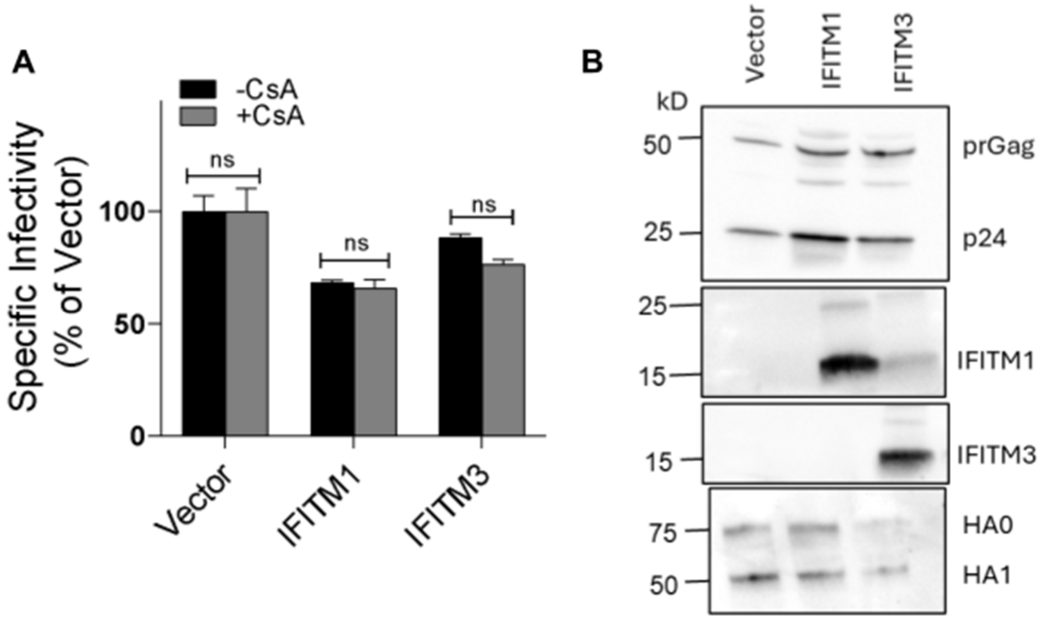

Figure S12. Effect of CsA on infectivity of IFITM-containing IAV pseudoviruses. (A) TZM-bl cells were infected by pseudoviruses bearing the Influenza A virus HA and NA and either IFITM1 or IFITM3. (B) Virus production, maturation and IFITM was verified by blotting for processed p24, IAV HA, and IFITM1 and IFITM3 proteins. See also Figure 2H, I.

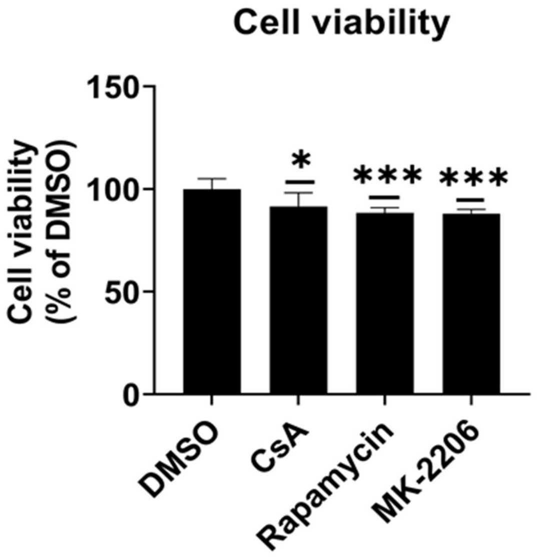

Figure S13. Effects of CsA, rapamycin, and MK-2206 treatment on A549 cell viability. Data represents cell viability of A549.vector cells treated with DMSO, CsA (20 µM), rapamycin (20 uM) or MK-2206 (10 µM) for 90 minutes. * $p < 0.05$; *** $p < 0.001$; ns, not significant. See also Figure 5.

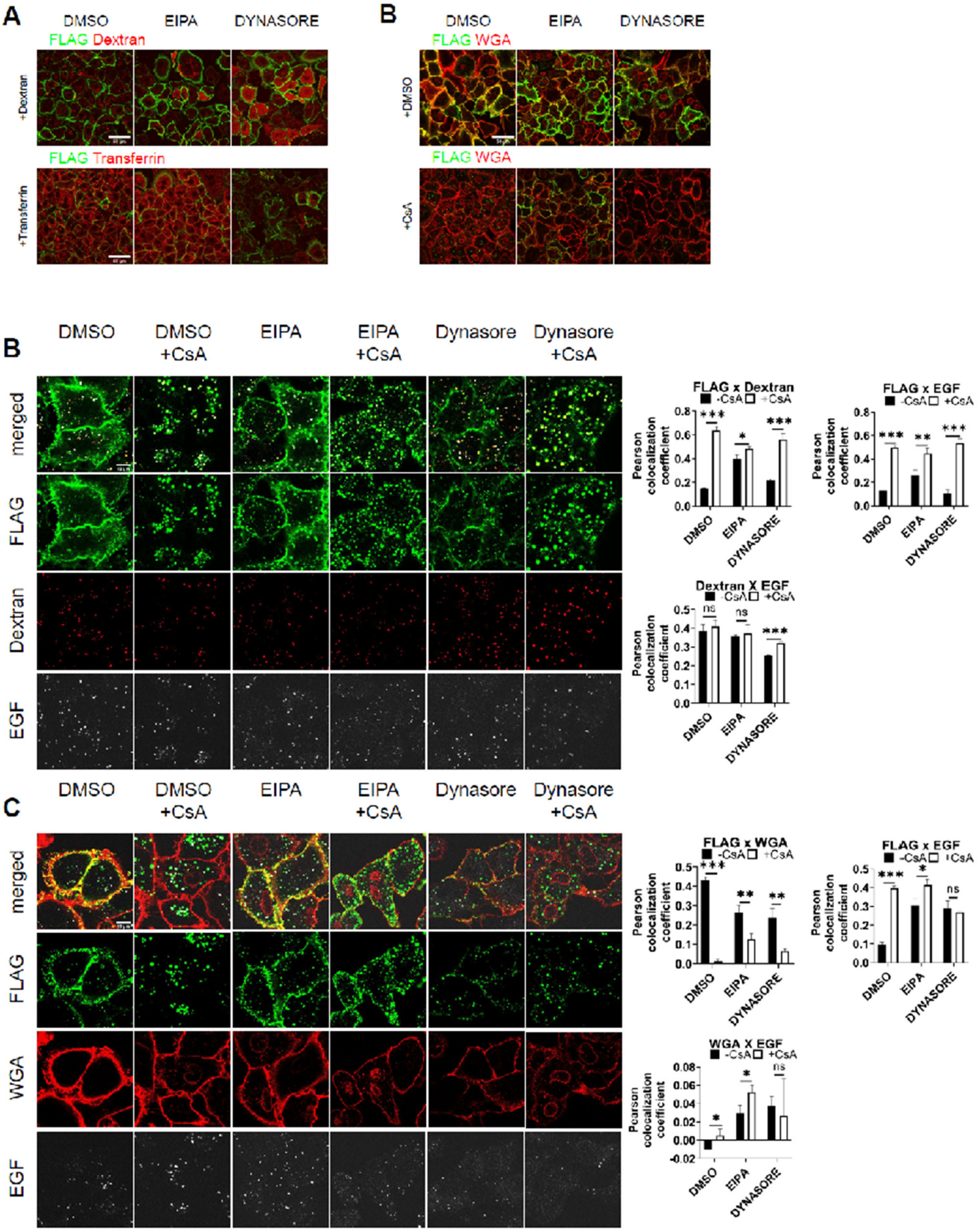
A
DMSO
EIPA
DYNASORE
FLAG Dextran
+Dextran
FLAG Transferrin
+Transferrin
B
DMSO
EIPA
DYNASORE
FLAG WGA
+DMSO
FLAG WGA
+CsA
B
DMSO
DMSO +CsA
EIPA
EIPA +CsA
Dynasore
Dynasore +CsA
merged
FLAG
Dextran
EGF
FLAG x Dextran
-CsA +CsA
Pearson colocalization coefficient
FLAG x EGF
Dextran X EGF
DMSO
EIPA
DYNASORE
C
DMSO
DMSO +CsA
EIPA
EIPA +CsA
Dynasore
Dynasore +CsA
merged
FLAG
WGA
EGF
FLAG x WGA
FLAG x EGF
WGA X EGF

Figure S14. CsA induces redistribution of IFITM1 from the plasma membrane predominantly via dynamin-independent pathway. (A) A549.IFITM1-C-Flag cells were pre-treated with the respective compounds (EIPA 50 µM, Dynasore 120 µM) before exposure to cargo (EIPA for 30 min, Dynasore for 15 min, for details, see the Methods section) for the designated uptake pathway—Dextran (macropinocytosis) or transferrin (dynamin-dependent endocytosis)—to assess inhibition. The integrated intensity and colocalization with the plasma membrane marker, IFITM1-C-Flag, were quantified and plotted. Scale bar 10 µm. (B) A549.IFITM1-C-Flag cells were pre-treated with the appropriate inhibitor and incubated with fluorescently tagged EGF on ice. The medium was replaced with a fresh medium containing dextran and either CsA or DMSO, with inhibitors maintained throughout. After 30 minutes, cells were fixed and imaged. The colocalization of Flag (IFITM1) with the respective markers (dextran, EGF) and between the markers was analyzed, and integrated intensity was measured. Scale bar 10 µm. (C) A549.IFITM1-C-Flag cells were treated as described in (B), but dextran treatment was omitted. Instead, the plasma membrane was stained with WGA post-fixation. The colocalization of FLAG (IFITM1) with the respective markers (WGA, EGF) and between the markers was analyzed, and integrated intensity was measured. For details, see the Methods section.

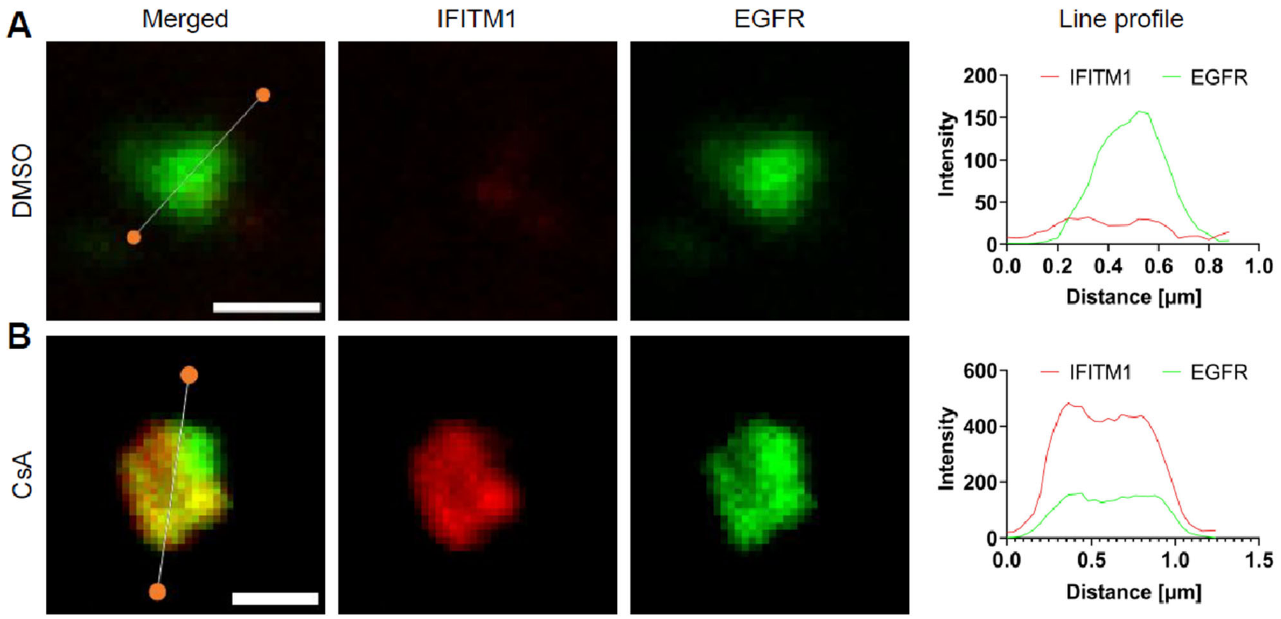

Figure S15. Redistribution of IFITM1 from the plasma membrane to the late endosome. A549.IFITM1-C-FLAG cells were treated with CHX, EGF and either DMSO (A) or CsA (B), as in Fig. 4B. Cells were fixed, permeabilized with TX-100, incubated with anti-IFITM1 and anti-EGFR antibodies, and stained with secondary antibodies conjugated to STED-compatible fluorophores, STAR RED and STAR 580. Representative images of n>20 analyzed endosomes are shown. Line histograms for selected endosomes are shown. Scale bar is 0.5 µm.

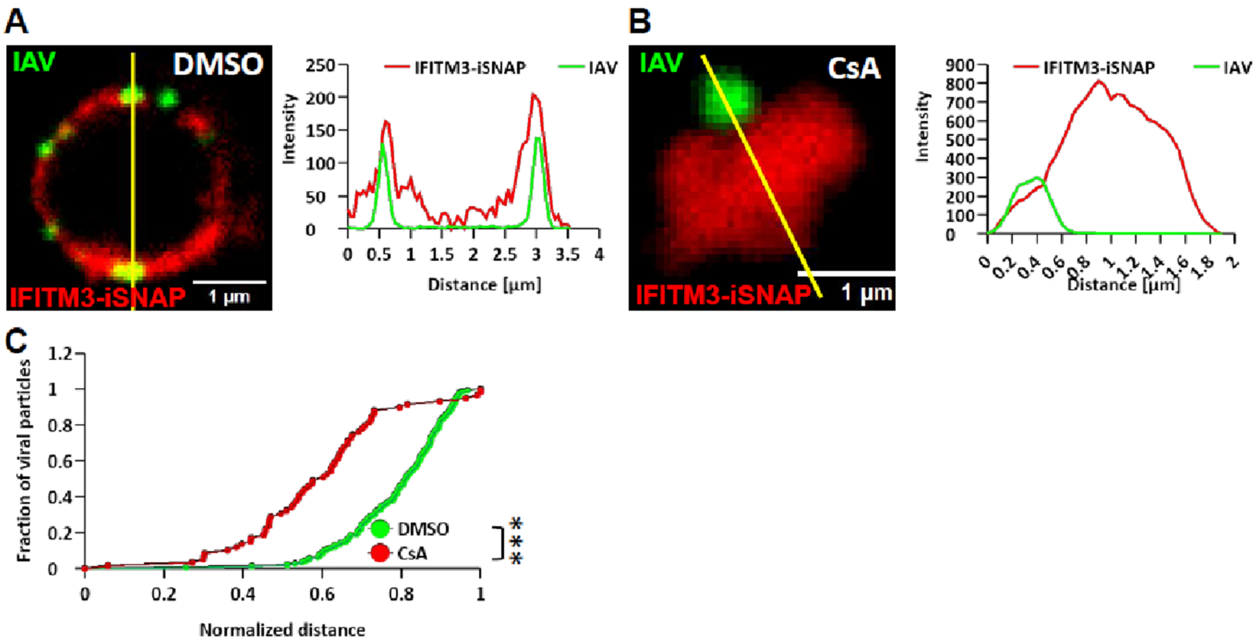

Figure S16. CsA induces redistribution of IFITM3 to the interior of late endosomes. (A-C) A549.IFITM3-iSNAP cells were pre-incubated with DMSO (A) or 20 µM of CsA (B) for 1.5 hours and spin-infected with AF-568 labeled IAV at MOI of 2. Infection was allowed to proceed for 1 hour in the presence of DMSO or CsA, at which time, cells were stained with SNAP-Cell 647-SiR for 30 min, washed and incubated with fresh medium for additional 30 min to remove unbound dye. Cells were fixed and imaged using STED super-resolution microscopy. Right graphs in panels A and B show the line intensity profiles across the endosomes and IAV particles corresponding to images on the left. (C) The distance of individual IAV particles to the center of the endosome was measured and normalized to the endosome's radius. Distances for IAV from at least 5 endosomes were measured and plotted for each condition. Lines and bars are means and S.D. ***, p < 0.001.

Movie S1. Staining of IFITM1 in the presence of DMSO. A549.IFITM1-C-FLAG cells were stained with anti-Flag antibody conjugated with AF-647 to visualize IFITM1 (green) and Hoechst to visualize nuclei (blue) and imaged in the presence of DMSO (vol %?) for indicated time. Time is in a mm:ss format. Movie is related to Figure S11.

Movie S2. Staining of IFITM1 in the presence of CsA. A549.IFITM1-C-FLAG cells were stained with anti-Flag antibody conjugated with AF-647 to visualize IFITM1 (green) and Hoechst to visualize nuclei (blue) and imaged in the presence of 25 µM CsA for indicated time. Time is in a mm:ss format. Movie is related to Figure S11.
